# Cardiac Tumors: Diagnosis, Prognosis, and Treatment

**DOI:** 10.1007/s11886-020-01420-z

**Published:** 2020-10-10

**Authors:** Rossana Bussani, Matteo Castrichini, Luca Restivo, Enrico Fabris, Aldostefano Porcari, Federico Ferro, Alberto Pivetta, Renata Korcova, Chiara Cappelletto, Paolo Manca, Vincenzo Nuzzi, Riccardo Bessi, Linda Pagura, Laura Massa, Gianfranco Sinagra

**Affiliations:** 1 Cardiothoracic Department, Institute of Pathological Anatomy and Histology, Azienda Sanitaria Universitaria Giuliano Isontina (ASUGI), University of Trieste, Via P. Valdoni 7, 34100 Trieste, Italy; 2 Cardiovascular Department, Azienda Sanitaria Giuliano Isontina (ASUGI), University of Trieste, Trieste, Italy

**Keywords:** Cardiac tumors, Masses, Multimodality imaging, Cardio-oncology, Histopathology, Neoplastic tumors

## Abstract

**Purpose of Review:**

Cardiac masses frequently present significant diagnostic and therapeutic clinical challenges and encompass a broad set of lesions that can be either neoplastic or non-neoplastic. We sought to provide an overview of cardiac tumors using a cardiac chamber prevalence approach and providing epidemiology, imaging, histopathology, diagnostic workup, treatment, and prognoses of cardiac tumors.

**Recent Findings:**

Cardiac tumors are rare but remain an important component of cardio-oncology practice. Over the past decade, the advances in imaging techniques have enabled a noninvasive diagnosis in many cases. Indeed, imaging modalities such as cardiac magnetic resonance, computed tomography, and positron emission tomography are important tools for diagnosing and characterizing the lesions. Although an epidemiological and multimodality imaging approach is useful, the definite diagnosis requires histologic examination in challenging scenarios, and histopathological characterization remains the diagnostic gold standard.

**Summary:**

A comprehensive clinical and multimodality imaging evaluation of cardiac tumors is fundamental to obtain a proper differential diagnosis, but histopathology is necessary to reach the final diagnosis and subsequent clinical management.

## Introduction

Cardiac masses frequently present significant diagnostic and therapeutic clinical challenges and encompass a broad set of lesions that can be either neoplastic or non-neoplastic. Moreover cardiac tumors may be symptomatic or found incidentally during evaluation for a seemingly unrelated problem or physical finding. Cardiac tumors represent a heterogeneous group, potentially involving any of the heart structures. We sought to provide an overview of cardiac masses focusing on each cardiac chamber and providing epidemiology, clinical presentation, imaging, histopathology, diagnostic workup, treatment, and prognoses of cardiac masses.

## Epidemiology and Classification

In 2015, the World Health Organization (WHO) updated the classification of cardiac neoplasms including benign tumors, tumor-like lesions, malignant tumors, and pericardial tumors [1]. Cardiac tumors are divided into primary and secondary forms.

The estimated prevalence for primary cardiac tumors is 1:2000 autopsies and for secondary tumors 1:100 autopsies, with a secondary/primary ratio of 20:1.

Approximately 10% of primary cardiac tumors are malignant and 90% benign (mostly myxomas) [2]. Myxomas account for approximately 50% of all benign cardiac tumors in adults and only for a small percentage in children. Rhabdomyoma is the most common benign tumor in children, accounting for 40 to 60% of the cases. Other benign cardiac tumors that have been described include fibromas, lipomas, hemangiomas, papillary fibroelastomas, cystic tumors of the atrioventricular node, and paragangliomas [2]. The remaining 10–20% of primary cardiac tumors are malignant and usually are pathologically described as sarcomas [3].

Primary cardiac sarcomas constitute approximately 1% of all soft tissue sarcomas and are the most common malignant primary cardiac tumor [4]. Angiosarcomas and unclassified sarcomas account for approximately 76% of all cardiac sarcomas, of which angiosarcomas are the most common [4]. Rhabdomyosarcoma is the most common form of cardiac sarcoma in children. Leiomyosarcoma, synovial sarcoma, osteosarcoma, fibrosarcoma, myxoidsarcoma, liposarcoma, mesenchymal sarcoma, neurofibrosarcoma, and malignant fibrous histiocytoma are other cardiac sarcomas observed [5].

As already mentioned, primary cardiac tumors are extremely uncommon (various postmortem studies report rates between 0.001 and 0.28%). Conversely, secondary tumors are more frequently encountered since the heart can theoretically be a site of metastasis by any malignant neoplasm [6••]. The exact incidence of cardiac metastatic disease is unknown. Bussani et al. from 1994 to 2003 performed 18,751 postmortem studies in the Pathologic Anatomy Department, University of Trieste. In 7289 cases, one or more malignant neoplasms were found (38.8% were evident at autopsy only), and 622 cases of heart metastasis were ascertained, resulting in an incidence of 9.1% of all malignant tumors [6••].

## Clinical Presentation

Clinical presentation of cardiac masses depends of the size, location, propensity for embolization, invasiveness, and relation with other cardiac structures. Some intracavitary cardiac tumors as lipomas are frequently asymptomatic, whereas others, like myxomas, represent the paradigm of clinical presentation: symptoms are mostly related to location, morphological characteristics, and cytokine production (particularly IL-6) resulted from mitral valve obstruction which may cause syncope, dyspnea, and pulmonary edema followed by embolic manifestations [7]. Patients may also present with nonspecific symptoms such as fatigue, cough, fever, arthralgia, myalgia, weight loss, erythematous rash, and laboratory findings of anemia, an increased erythrocyte sedimentation rate, and increased levels of C-reactive protein and gamma globulin. Dyspnea that worsens lying on the left side should orient clinicians towards the possibility of a myxoma. Less common findings are thrombocytopenia, clubbing, cyanosis, or Raynaud phenomenon [8]. Physical examination might disclose an early diastolic sound (“tumor plop”) for atrial myxomas with valvular prolapse [8].

In other case, as for sarcomas, patients typically present with advanced disease, and 66–89% of patients already have evidence of metastatic disease at clinical presentation. Patients with a cardiac sarcoma often present with dyspnea, atypical or pleuritic chest pain, syncope, presyncope, and fatigue [9].

In case of intramural masses, symptoms are associated with conduction disturbances and arrhythmias or sudden cardiac death, and, as for fibroma, symptoms may be related to the growth of the mass which may cause vascular obstruction and heart failure [10]. Usually hamartomas, tumors affecting mainly young children, can present with unremitting ventricular tachycardia [11].

Finally, as in primary lymphomas, patients could present with systemic symptoms (fever, sweats, and weight loss) and symptoms related to pericardial effusion [12].

The electrocardiogram (ECG) can show a number of abnormalities, including evidence of left ventricular hypertrophy, right ventricular hypertrophy, bundle branch block, atrioventricular block, and ventricular tachycardia for fibromas, whereas myxomas commonly show left atrial enlargement or nonspecific findings [10].

**Fig. 1:**
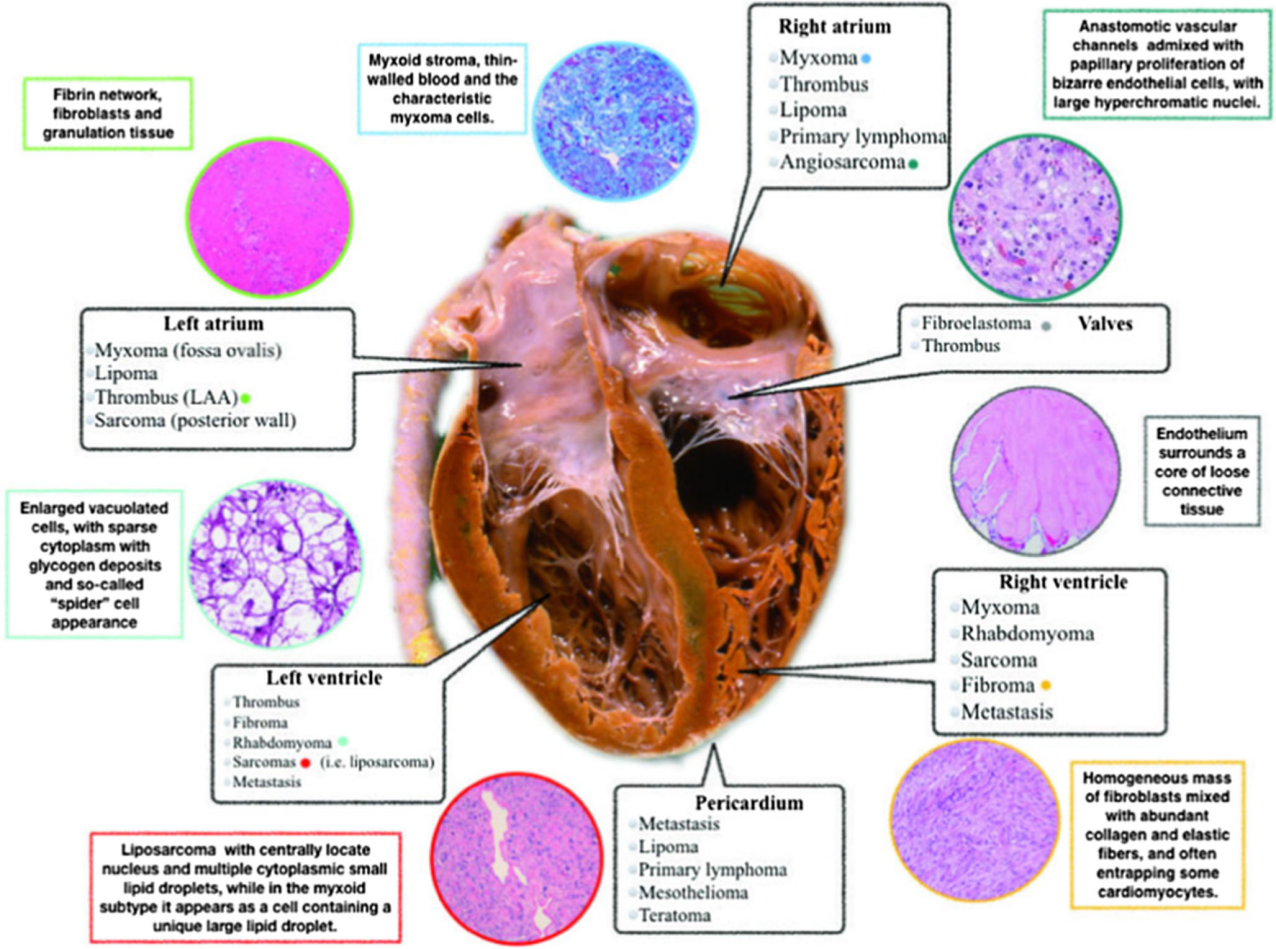
Overview of localization of cardiac tumors. (From Castrichini et al. European Heart Journal - Case Reports. doi:10.1093/ehjcr/ytaa026,by permission of Oxford University Press) [13••]

Cardiac tumors may arise from any part of the heart (Fig. 1); however, myxomas are found predominantly in the left atrium, lipomas tend to occur in right atrium or in the left ventricle, and fibroma and rhabdomyomas are mostly located in the ventricle. Finally, angiosarcomas occur most commonly in the right atrium, whereas undifferentiated pleomorphic sarcomas occur in the left atrium. Although cardiac tumors could develop in any chambers, each cardiac tumor may be more frequent in one of them; therefore, a cardiac chambers approach may be useful.

## The Left Atrium

### Myxoma

Cardiac myxoma is the most common type of benign primary cardiac neoplasm and can be present at any age but most often between the fourth and sixth decades of life, with a slight female predominance (1.5:1) among individuals aged < 65 years [14]. More than 80% of them are found in the left atrium, but they are also found less frequently in the right atrium, right ventricle, and left ventricle [3]. The exact origin of myxoma cells remains uncertain, but they are thought to arise from remnants of subendocardial cells or multipotential mesenchymal cells in the region of the fossa ovalis. Most myxomas occur sporadically, but they may be familial and occasionally (3–10%) related to a particular syndrome called “the Carney complex,” an autosomal dominant condition characterized by endocrinopathy (Cushing syndrome or acromegaly) and spotty skin pigmentation [14]. The Carney complex occurs at a younger age and should be considered when cardiac myxomas are discovered in atypical locations in the heart. This disease results from inactivating mutations in genes encoding cAMP-dependent protein kinase type 1α regulatory subunit (PRKAR1A) [14].

#### Imaging

Transthoracic echocardiography (TTE) and transesophageal echocardiography (TEE) can be used in combination to assess tumor size, shape, morphology, and hemodynamic effects [15]. Cardiac myxomas are usually attached by a stalk to the atrial septum in the fossa ovalis and have lobulated margins, and their range of movement is dependent on stalk length, size, and morphology. Cardiac myxomas will enhance with use of echocardiography contrast [15]. On computed tomography (CT), a myxoma appears as a well-defined, ovoid, intracavitary mass with lobulated contours, and the contrast helps to delineate the mass as a low-attenuation lesion surrounded by enhanced intracardiac blood [16]. On cardiac magnetic resonance (CMR) images, they appear isointense on T1-weighted sequences and have higher signal intensity on T2-weighted sequences owing to the high extracellular water content [17]. Regions of acute hemorrhage appear hypointense on both T1-weighted and T2-weighted images and can subsequently become hyperintense as hemoglobin in the blood is progressively oxidized [17]. Steady-state free precession imaging can reveal the stalk-like attachment and the mobile nature of these masses, as well as prolapse across valves. Internally, myxomas may contain cysts, regions of necrosis, fibrosis, hemorrhage, and calcification, which lead to a typically heterogeneous appearance at contrast enhancement. Postcontrast delayed imaging typically shows a heterogeneous enhancement pattern, with many myxomas having a layer of surface thrombus with low signal intensity [17].

#### Histopathology

Traditionally, myxomas are yellowish, white, or brownish pedunculated masses frequently covered with thrombus at gross examination. The tumor size can range from 1 to 10 cm showing a smooth surface in most cases [18]. A villous or papillary form of myxoma has been reported with a surface consisting of multiple fine or very fine villous, gelatinous, and fragile extensions that have a tendency to fragment spontaneously and are associated with embolic phenomena [8]. Histologically, the tumors are characterized by lepidic cells in a myxoid stroma, Alcian-PAS positive, usually with degenerative features such as calcification and hemorrhage, and the abundance of myxoid stroma is also associated with tumor embolization, in which the tumor breaks off and travels downstream, causing a blockage [18]. Immunohistochemical investigation of PRKAR1A is a potential screening tool to evaluate whether myxomas develop in the setting of Carney complex [19].

### Undifferentiated Pleomorphic Sarcoma

Undifferentiated pleomorphic sarcomas are the most frequent primary cardiac malignancy (10% of all primary cardiac tumors). They occur mostly in the left atrium but can develop in any chamber [20], and they occur mainly in adults, with no sex predilection.

#### Imaging

Undifferentiated sarcomas appear as large, irregular, intracavitary masses on CT with low-attenuation [20], which usually appear isointense on T1-weighted images and hyperintense on T2-weighted images, with a heterogeneous, delayed enhancement pattern [17, 21].

#### Histopathology

**Fig. 2:**
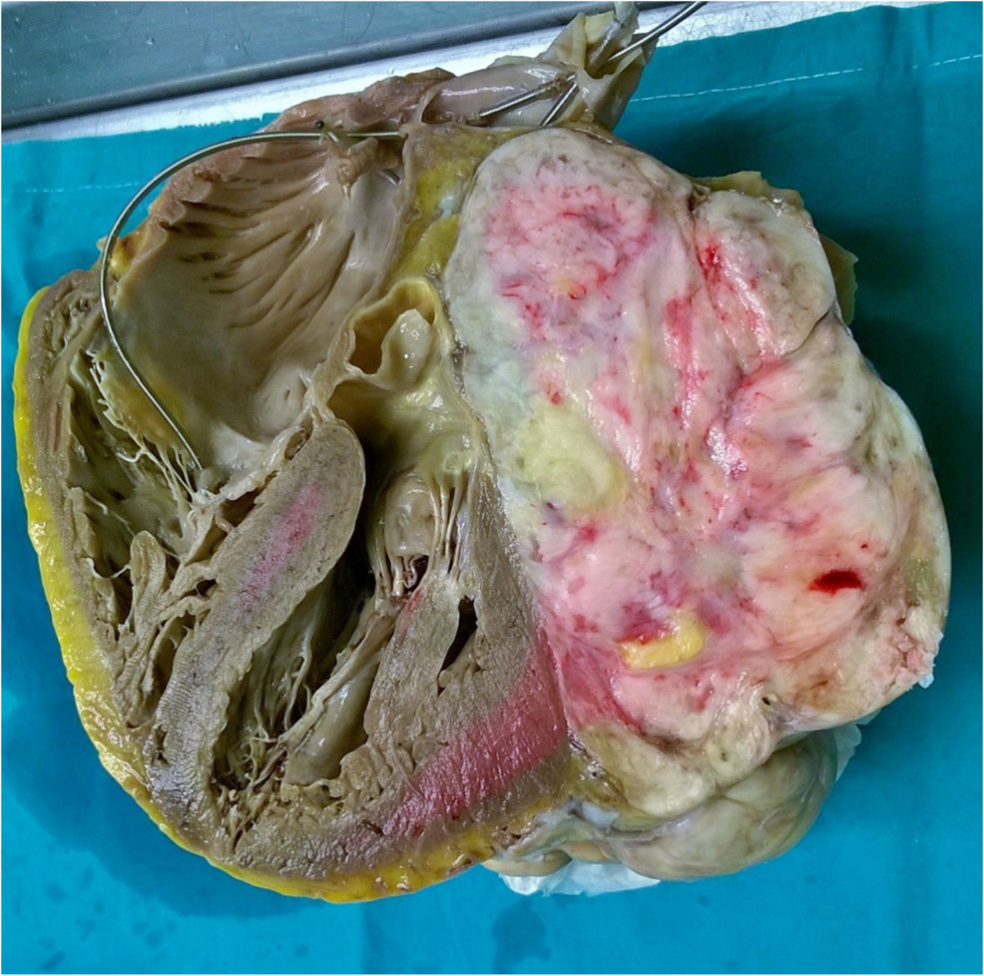
Metastatic pleomorphic sarcoma. We can see a large neoplastic mass extensively necrotic area destroying the subtotality of the left atrium

**Fig. 3:**
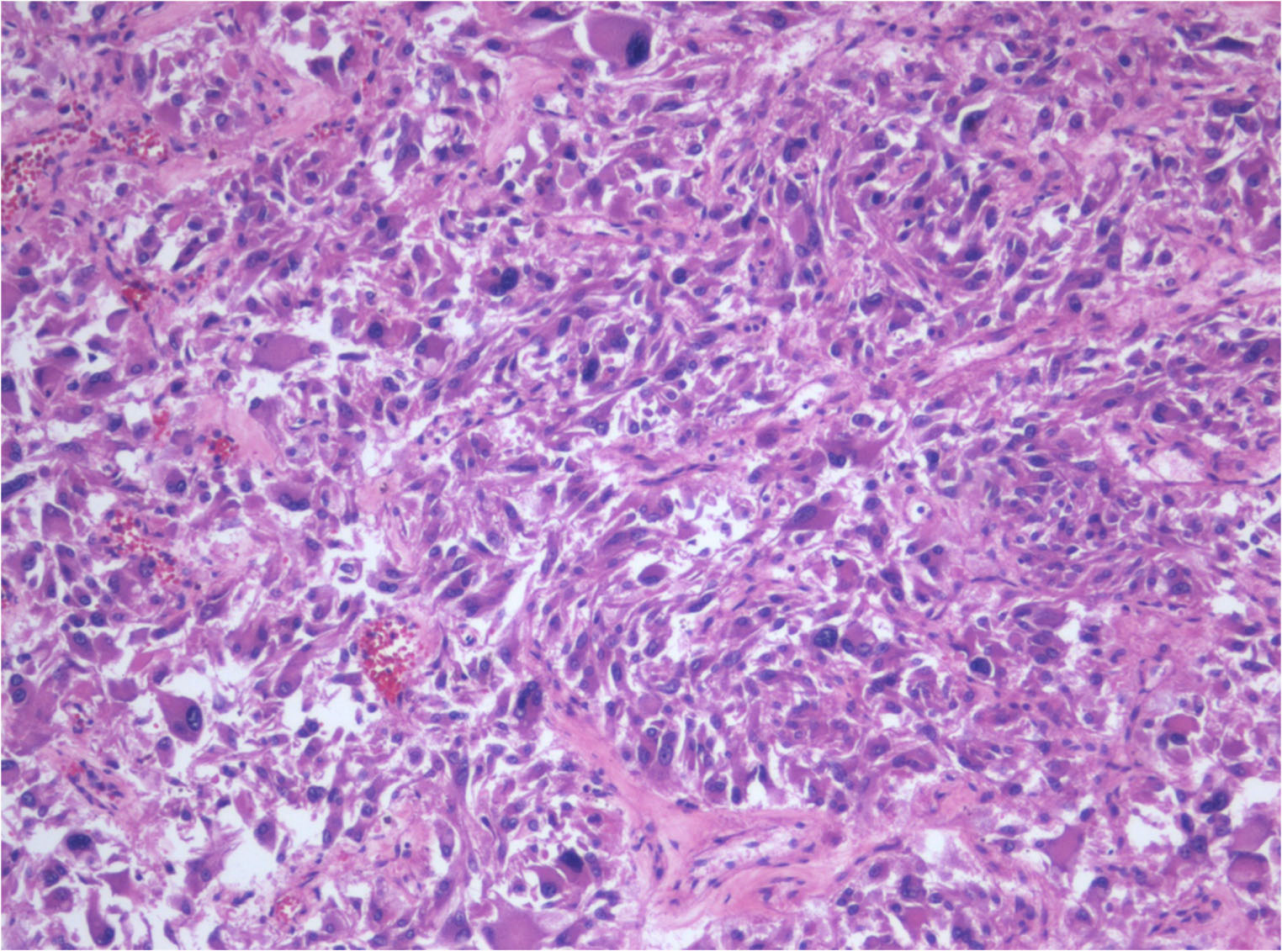
Metastatic pleomorphic sarcoma (EE × 10). The tumor consists of pleomorphic and anaplastic cells, both with spindle pattern and epithelioid cells

A definite confirmation requires either immunohistochemical or ultrastructural confirmation. Diagnostic criteria include the presence of typical spindle and polygonal (strap-like) cells that are filled with an abundant eosinophilic cytoplasm and, particularly, desmin- and myoglobin-positive immunoreactivity (Figs. 2, 3). On immunohistochemistry, these cells are CD68 negative [20].

## The Right Atrium

### Lipoma

Lipoma is a rare and mostly sporadic benign cardiac tumor; it makes up only 3% of all benign tumors. Lipomas tend to occur in the right atrium but may be found anywhere in the heart, as well as the pericardium. Lipomas are associated with older age, increased body mass, and female sex [14].

#### Imaging

The echocardiographic appearance of lipomas varies with their location. Lipomas in the pericardial space may be completely hypoechoic, have hypoechoic regions, or be completely echogenic, whereas intracavitary lipomas are homogeneous and hyperechoic [22]. A CT scan can provide better tissue characterization because cardiac lipomas display a low-attenuation signal similar to subcutaneous or mediastinal fat [22]. On CMR, these lesions have similar signal intensity to the surrounding fat on the chest wall on T1-weighted and T2-weighted images. Homogenous high signal intensity relative to the myocardium on T1-weighted images that is markedly suppressed with the application of fat saturation prepulses is indicative of the presence of a lipoma [17]. Given their avascularity, these lesions do not enhance on delayed postcontrast imaging.

#### Histopathology

Grossly, cardiac lipomas usually occur as single, well-encapsulated masses, though multiple lesions can occur. A cardiac lipoma is histopathologically composed of mature fat cells with occasional fibrous connective tissue and vacuolated brown fat. The differential diagnosis with lipomatous hypertrophy is important and crucial. Septal thickening is usually limited to the limbus of the fossa ovalis. Lipomatous hypertrophy, histologically, is a non-encapsulated lesion that contains three elements: mature fat, immature fat, and atrial myocytes. In lipomas, areas of degeneration and extensive radiographically apparent calcification may be present.

### Angiosarcoma

Cardiac angiosarcomas, malignancies of endothelial cells, are the most common primary differentiated cardiac neoplasms. They predominantly affect individuals aged 40–50 years but have been described in all age groups [22]. They occur most commonly in the right atrium but can develop in any chambers [23].

#### Imaging

On echocardiography, angiosarcomas appear as a dense and irregular mass, often nonmobile and broad-based, with endocardial to myocardial extension. On CT imaging, a low-attenuation, irregular, intracavitary mass is often shown, mostly involving the right atrial free wall. Given the vascular nature of the lesion, angiosarcomas show a heterogeneous enhancement pattern with intravenous iodinated contrast [24]. On CMR, heterogeneous T1- and T2-weighted signal intensity patterns reflect tumor tissue, necrosis, and hemorrhage; arterial phase enhancement at first-pass perfusion owing to vascularity; and heterogeneous enhancement at LGE imaging owing to peripheral fibrosis (surface hyperintensity) and focal hypointensity due to central necrosis [24].

#### Histopathology

Macroscopically, there are two morphologic variants: The “focal” variety is typically a well-defined mass protruding into the right atrium, causing serious intracavitary obstruction; the “diffuse” variety is a more extensive mass that rapidly infiltrates the right ventricle and pericardium that manifests with right-sided heart failure or tamponade [21]. Microscopically, angiosarcomas consist of rapidly proliferating, extensively infiltrating anaplastic cells derived from blood vessels and lining irregular blood-filled spaces, and there are usually large regions of hemorrhage and necrosis within the tumor [21].

### Lymphoma

Primary cardiac lymphomas are extranodal lymphomas that primarily involve the heart and/or the pericardium. They can arise in both immunocompetent and immunocompromised individuals. Men are affected twice as often as women, with most patients aged > 60 years [12]. Echocardiography will often show pericardial effusion, despite CT imaging might be more accurate for staging purposes. On CT imaging and CMR imaging, the findings are relatively nonspecific. Cardiac lymphomas are typically homogenous and isointense on T1-weighted and T2-weighted images and hypermetabolic on positron emission tomography (PET) imaging [21]. The majority of patients have diffuse large B cell non-Hodgkin lymphomas, usually expressing CD20 [12]. Treatment options for this subtype include monoclonal antibody rituximab treatment, alongside a regimen of four chemotherapy drugs (cyclophosphamide, doxorubicin, prednisone, and vincristine) [10]. Cardiac lymphomas are associated with an ominous outcome.

## The Ventricles

### Fibroma

Cardiac fibromas are benign proliferations and are the second most common intracardiac tumor in children, having in some cases congenital origins, but they can also occur in adults [14]. Most often, a fibroma is located in the ventricle and interventricular septum, and patients may present with chest pain, pericardial effusion, heart failure, or arrhythmias [25, 26]. Cardiac fibromas are associated with Gorlin syndrome, an autosomal dominant disease caused by mutations in the *PTCH1* gene that is characterized by nevoid basal cell carcinoma, unusual brain tumors, skeletal abnormalities, and macrocephaly [25]. As rhabdomyomas, it can be detected in the ventricles (predominantly in the left ventricle) of fetuses, children, and (rarely) adults [14].

#### Imaging

On echocardiography, cardiac fibromas are homogeneous, appearing brighter than surrounding myocardium, and might incorporate hyperintense flecks suggestive of calcium [18]. On CT imaging, fibromas appear as solitary, intramural, homogenous masses with soft tissue attenuation that can be either sharply demarcated or infiltrative, alongside areas of calcification [16]. With CMR imaging, fibromas are isointense relative to normal myocardium on T1-weighted images and are characteristically hypointense on T2-weighted images (unlike other masses). They are generally homogeneous unless there is central calcification, which may be seen as patchy central hypointensity. With gadolinium-based contrast agent administration, fibromas generally show no contrast enhancement during perfusion imaging due to avascularity. However, after 7–10 min, they classically show intense hyperenhancement on late gadolinium enhancement images [27]. This phenomenon is likely to be attributable to the collagenous nature of these lesions.

#### Histopathology

Cardiac fibromas appear well-circumscribed and manifest as tan nodules with a whorled appearance, not unlike scar tissue. Despite the well-circumscribed gross appearance, they tend to extend into adjacent myocardium. Fibromas are histologically composed primarily of fibroblasts or collagen, with the typical “spindle cells.” Calcification of fibromas is seen more commonly in older individuals and can help to differentiate these tumors from cardiac rhabdomyomas [12, 21–24, 28].

### Rhabdomyoma

Rhabdomyomas are the most common benign cardiac tumor in children and arise most commonly in the ventricles [21]. Approximately 80% of them are found in association with tuberous sclerosis [29] that is characterized by the diagnostic triad of seizures, mental retardation, and facial angiofibromas [14]. Almost 34% of these patients will be diagnosed with rhabdomyomas [30, 31].

#### Imaging

On echocardiography, rhabdomyomas appear as a bright ventricular mass protruding, or not, into the chamber. On cardiac MRI, these lesions are isointense/normal on T1-weighted images but hyperintense on T2-weighted images and typically have minimal delayed enhancement [32, 33].

#### Histopathology

Cardiac rhabdomyomas are composed of enlarged, vacuolated cardiomyocytes and are characterized by typical spider cells, polygonal myocytes with prominent sarcoplasmic clearing.

### Hemangioma

Hemangiomas are more frequently seen and account for 2% of primary cardiac neoplasms. Hemangiomas can occur in any age group ranging from a few months to the seventh decade of life [12]. Cardiac hemangiomas are considered to be benign neoplasms with the potential for recurrence, but the etiology is not defined. They can occur in any cardiac chamber, although they occur more frequently in the ventricles and in about 30% of cases are multiple [22].

#### Imaging

Cardiac hemangiomas appear as hyperechoic lesions, in the 25% of cases projecting in the cavity and, on CT and CMR, are characterized by contrast enhancement [21, 22].

#### Histopathology

Histologically, it could be characterized by small capillaries (capillary hemangioma) or large vessels (cavernous hemangioma, the most common) or also might be dysplastic (cirsoid aneurysm) [34].

### Hamartoma of Mature Cardiac Myocytes

They occur mostly in men (2:1), the mean age is 25 years, and they have been implicated as a possible cause of sudden death [12].

#### Imaging

On echocardiography, it appears as a homogeneous, intracardiac, poorly circumscribed mass that seems a myocardial scar. Histologically, the lesions are characterized by a nodular collection of enlarged, highly disorganized cardiomyocytes [10, 12, 25–27, 29–34].

## The Valves

### Fibroelastoma

**Fig. 4:**
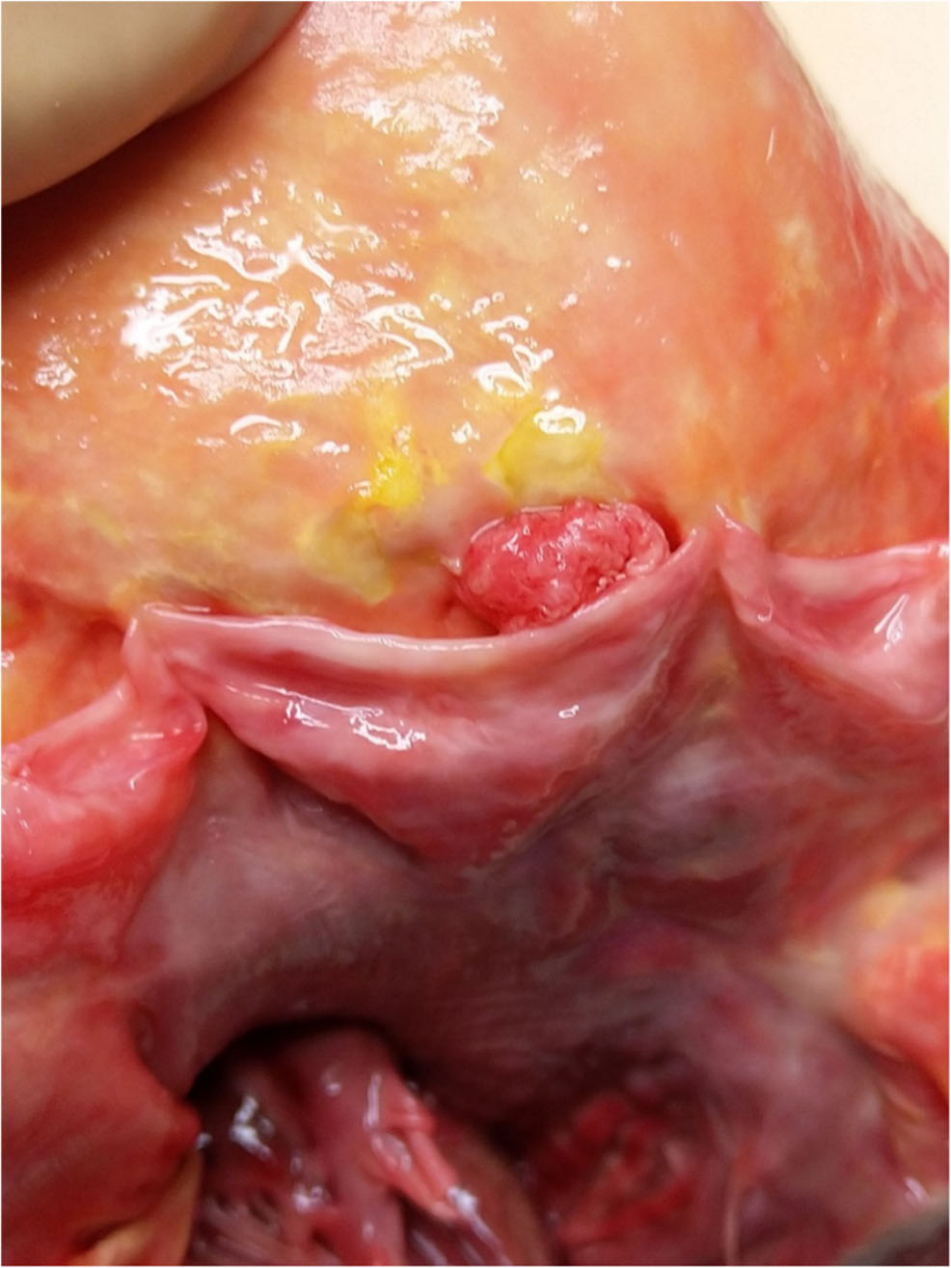
Giant papillary fibroelastoma occluding the right coronary artery ostium

Papillary fibroelastomas are benign, endocardial growths that mostly involve valvular tissue, in particular the aortic valve (Fig. 4). Mean age is 70–80 years old with no sex differences [35]. Papillary fibroelastomas are morphologically similar to Lambl’s excrescences, without complex branching, and occur mainly on valvular closing surfaces [21].

#### Imaging

On echocardiography, papillary fibroelastomas are usually pedunculated masses with independent mobility and homogeneous speckled pattern [36]. On CT and CMR, papillary fibroelastomas could be seen upstream or downstream from the valves but can also be present on other endothelial surfaces [22].

#### Histopathology

Papillary fibroelastomas range in size from 2 to 5 cm and if large could exhibit calcification and fibrotic matting of the papillary fronds. Microscopically consist of endocardium-coated fronds with an avascular collagenous core [4].

## The Conduction System

### Cystic Tumor of the Atrioventricular Node

The cystic tumor of the atrioventricular (AV) node involves the atrioventricular node selectively. It is also known as tawarioma or celothelioma (mesothelioma of the AV node) reflecting its controversial histogenesis [37]. The mean age of clinical presentation is nearly 40 years. A total of 75% of patients present with complete and 15% with incomplete AV block, whereas in 10%, sudden death is the first clinical manifestation [38].

#### Histopathology

The tumor appears multicystic, with size varying from 2 to 20 mm. Histologically, the tumor is located on the right side of the central fibrous body, infiltrating and compressing the AV node. The cysts are filled by a mucoid substance and are lined by epithelium, cytokeratin, and epithelial membrane antigen positive. Diagnosis is usually achieved at postmortem or after cardiac transplantation through histological examination of the conduction system but occasionally in vivo or during surgical resections [39].

## The Pericardium

### Malignant Mesothelioma

Mesotheliomas are malignant tumors that could rarely arise from the pericardium (1%) [44–25]. The relationship between them and asbestos exposure or radiotherapy has not yet been established [2–12, 14–44]. Mean age is 50–70 years old with a 2:1 male-female ratio [45]. These tumors produce nonspecific signs and symptoms or may lead to tamponade and constriction. The diagnosis of pericardial mesothelioma is usually difficult and late and, to date, only 25% of cases being diagnosed antemortem [46, 47].

#### Imaging

Chest radiograph typically shows enlarged cardiac silhouette with pericardial effusion.

Echocardiography is the most commonly used initial cardiac imaging modality and could be useful also in guiding pericardiocentesis. However, CT and CMR are generally required to provide additional information about size, location, and extent of pericardial involvement [12]. PPM can cause focal increased uptake of radiotracer on positron emission tomography/computed tomography scan (PET/CT) [47], which can be used in distinguishing whether a nodule or mass is benign or malignant.

#### Histopathology

Mesothelioma cells may have three distinct patterns, that is, predominantly epithelial, predominantly fibrous (spindle cell), and biphasic (mixed) [48]. Negative adenocarcinoma markers, such as carcinoembryonic antigen (CEA) and positive mesothelial markers (calretinin and cytokeratins), could be useful to differentiate mesotheliomas from pericardial metastasis of adenocarcinoma [48].

## Secondary Cardiac Tumors

In decreasing order, the tumors with the highest rate of heart metastasis are pleural mesothelioma (48.4%), melanoma (27.8%), lung adenocarcinoma (21%), undifferentiated carcinomas (19.5%), lung squamous cell carcinoma (18.2%), and breast carcinoma (15.5%). High rates of heart metastasis have also been observed in patients affected by ovarian carcinoma (10.3%), lymphomyeloproliferative neoplasms (9.4%), bronchioalveolar carcinomas (9.8%), gastric carcinomas (8%), renal carcinomas (7.3%), and pancreatic carcinomas (6.4%) [6••].

Cardiac involvement may occur via the blood stream, direct invasion from the mediastinum, or tumor growth into the vena cava and extension into the right atrium [12]. Lymphatic spread tends to give rise to pericardial metastasis, whereas hematogenous spread tends to give rise to myocardial metastasis [6••].

Clinical presentations of cardiac metastasis are extremely variable and differ greatly according to the most heavily involved site. Although a cardiac metastasis may be the first or even the only manifestation of an undiagnosed malignant tumor, they often go unrecognized in vivo and are diagnosed only after death [10, 12, 21–24, 28]. In the case of secondary tumors located in the myocardium, the clinical pattern will be proportional to the degree of myocardial infiltration or in some way related to the wall infiltration site [6]. Typical presentation includes arrhythmias and conduction disturbances and complete atrioventricular blocks, especially if the conduction system has been infiltrated [49].The diagnostic evaluation is the same as that for primary cardiac tumors and relies upon echocardiography, CMR imaging, and CT to ascertain the extent of cardiac involvement. However, the method of choice to detect cardiac metastasis and their complications is echocardiography [34]. Fluoro-D-glucose (18F-FDG) PET/CT is the main molecular imaging modality used to evaluate the malignant nature of the mass and staging and can be used to detect recurrence post-surgery and metastasis [12, 21–24, 28]. Heart metastasis can present a great variety of morphological aspects depending on tumor type, site, spreading capacity, and mode of permeating the heart [34]. Pericardial metastasis may present with focal, diffuse, or massive infiltration with or without metaneoplastic fibrin–blood effusion. Myocardial metastasis can involve any one of the heart chambers. Metastasis derivated by blood embolization may result in lesions of remarkable size, sometimes compressing the surrounding myocardium and causing secondary hypoperfusion [6••]. Neoplastic invasion secondary to lymphoma typically tends to replace the myocardial tissue, and broad heart areas are globally infiltrated by homogenized white grayish tissue, with the typical “fish meat” appearance [6••]. A standard treatment modality for cardiac metastasis has not yet been established. Because most patients with cardiac metastasis have disseminated disease, the therapy generally consists of treating of the primary tumor or palliative care. Despite the poor prognosis of patients with cardiac metastasis, however, surgical treatment should be considered when important symptoms of obstruction outweigh the mortality risk of operating and the benefit of medical therapy alone [6••].

## Diagnostic Workup

**Fig. 5:**
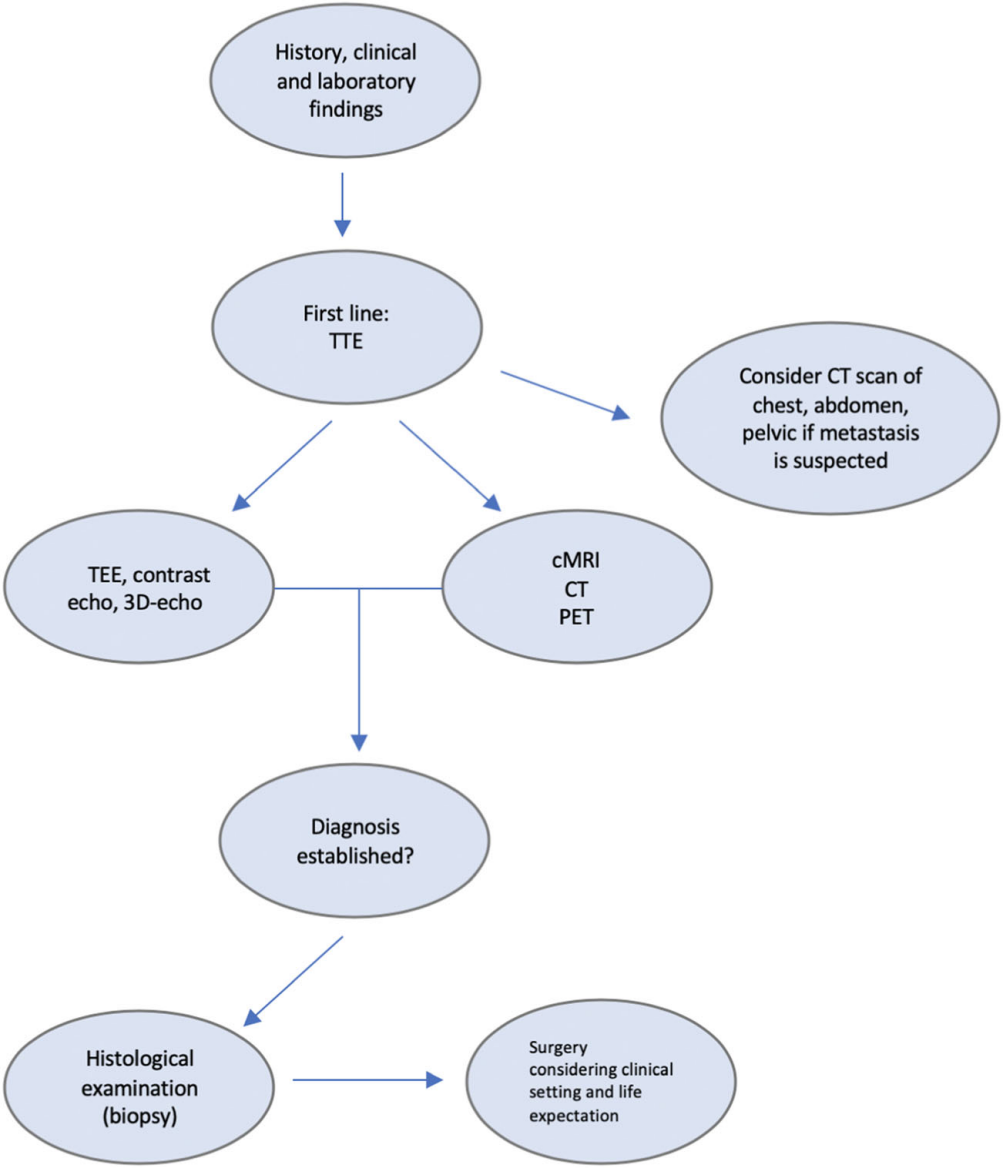
Flow chart of diagnostic workup

**Fig. 6:**
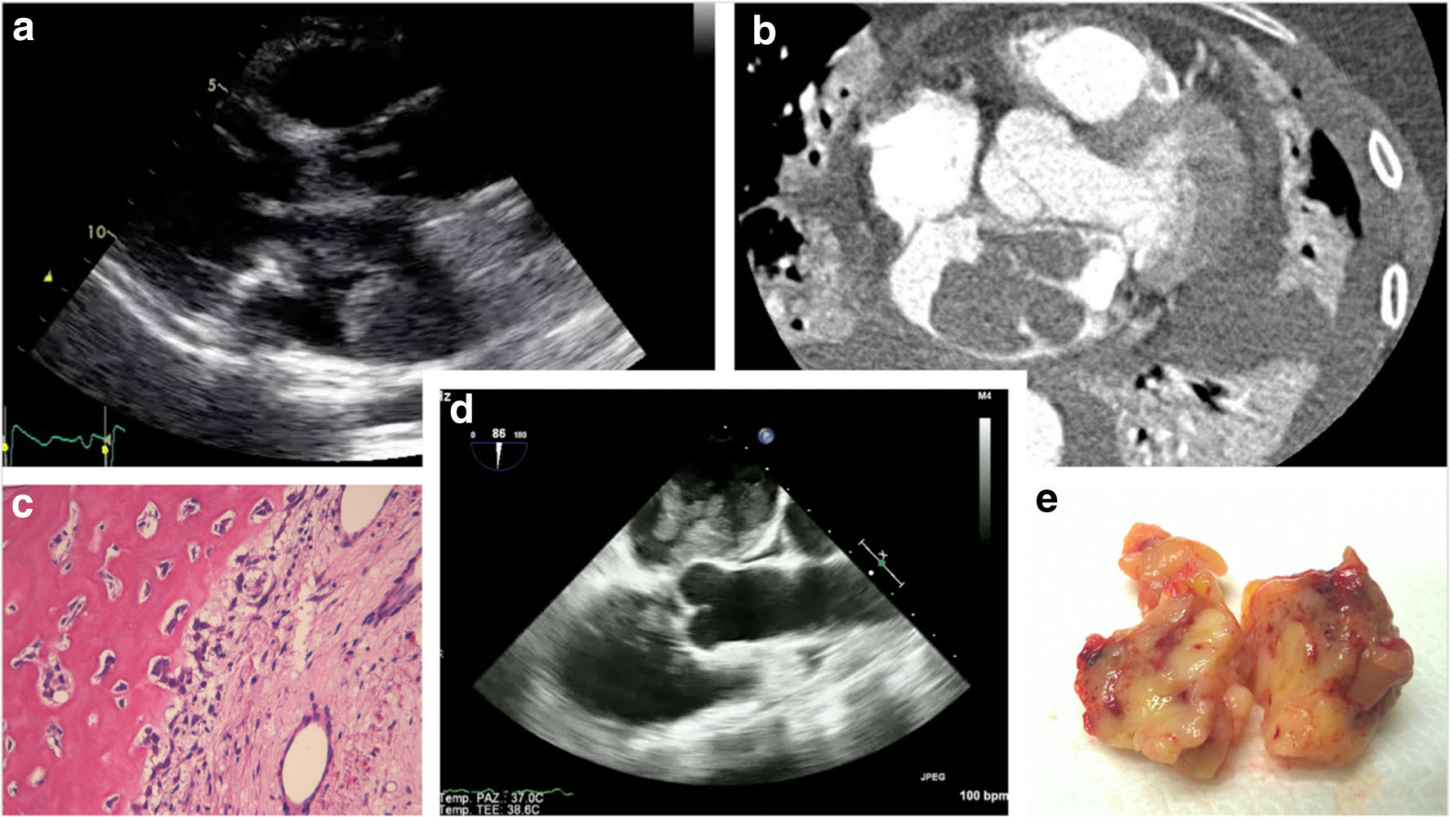
Giant liposarcoma in the left atrium. **a** TTE long axis section shown left atrium mass with homogenous echogenicity. **b** CT scan. **c** Dedifferentiated liposarcoma with osteogenic areas (EE × 20). **d** TEE long axis view. **e** Liposarcoma after surgical abscission

Every mass should be put in the clinical context, by collecting information of past medical history, age, gender, and laboratory tests before proceeding to imaging exams. A mass in a child is most probably a rhabdomyoma or a fibroma, while a patient with atrial fibrillation, ischemic heart disease, cardiomyopathy, or hypercoagulability may develop intracardiac thrombi [12]. Instead in a patient with native or prosthetic valve disease or endocavitary catheter, a mass requires differential diagnosis between tumor, vegetation, calcification, and thrombus. Finally, if patients with a malignancy develop cardiac symptoms or signs, the possibility of a secondary involvement should be considered. Cardiac masses are identified by multimodality noninvasive imaging. TTE remains the first diagnostic approach and allows evaluating size, contours, mobility, site of attachment, and hemodynamic impact of the mass [34]. Myxomas appear with finger-like projections or with a smooth surface and could present inhomogeneous areas of hyperechogenicity due to calcification; conversely thrombi have frequently homogenous echogenicity, usually are not highly mobile, and are often found in the atrial appendage and in the context of atrial fibrillation [50•]. CMR is the best available noninvasive diagnostic tool to provide information about topographic relations, extension to surrounding structures, tissue characterization, and specific patterns of enhancement (absent, early, or delayed) after contrast medium administration [21]. Contrast-TTE is useful to assess the perfusion of the mass in order to differentiate vascular tumor from thrombi. Malignant tumors are frequently highly vascularized and present greater contrast enhancement than the adjacent myocardium, whereas myxomas demonstrate partial perfusion on visual inspection and quantitatively less perfusion than the surrounding myocardium; thrombi, being avascular, show complete absence of perfusion [51]. In the complex field of cardiac masses, a multimodality imaging approach is crucial to reach the diagnosis. However, histologic examination is required in challenging scenarios [21](Fig. 5, Fig. 6).Histopathological characterization remains the diagnostic gold standard in any resected cardiac mass, allowing to establish the benign or malignant nature and the precise histotype [34] (Table 1).

**Table 1:** Relevant pathological features of most common cardiac tumors

		Epidemiology	Histology features	Echocardiography	CMR tissue characterization
Left atrium	*Myxoma*	-50% of all benign cardiac tumors in adults. -30–50 years of age. -Males > females -4.5–10% are familial	Spindle or stellate cells, pseudovascular structure, myxoid matrix, hemorrhages. dystrophic calcification can be present	-Narrow stalk -Fossa ovalis -Isoechogenic	-Iso- to hypointense to on T1, hyperintense on T2 -Heterogeneous enhancement with contrast
	*Undifferentiated pleomorphic Sarcoma*	-10% of all primary cardiac tumors. -45 years old -No sex predilection	-Typical spindle and polygonal (strap-like) cells filled with an eosinophilic cytoplasm -Desmin- and myoglobin-positive -CD68 negative	Broad-based mass with heterogeneous echogenicity	-Isointense on T1and hyperintense on T2-weighted images -Heterogeneous, delayed enhancement
Right atrium	*Lipoma*	-3% of benign tumors -Associated with older age, increased body mass and female sex	Mature fat cells with occasional fibrous connective tissue and vacuolated brown fat	Lipomas in the pericardial space may be completely hypoechoic, whereas intracavitary lipomas are homogeneous and hyperechoic	Hyperintense bright signal in T1 and T2, reduced with fat suppression technique No enhancement
	*Angiosarcoma*	The most common primary differentiated malignant neoplasms. -40–50 years old	Highly vascularized, myocardial infiltration, pleomorphism, necrosis and mitosis	Dense and irregular mass, often nonmobile, broad-based, with endocardial to myocardial extension	Heterogeneous in T1 and T2. Heterogeneous contrast enhancement (“sunray appearance”)
	*Lymphoma*	Most common in the pericardial space	Diffuse large B cell non-Hodgkin lymphomas, usually expressing CD20	Pericardial effusion	Isointense in T1 and T2 No/variable contrast enhancement
The ventricles	*Fibroma*	Second most common pediatric cardiac tumor	Fibroblasts and collagen bundles, some elastic fibers, calcification is a common finding	-Homogeneous, appearing brighter than surrounding myocardium -Might incorporate hyperintense flecks suggestive of calcium	Hypointense in all T1, T2 steady-state free procession Late contrast enhancement
	*Rhabdomyoma*	The most common benign cardiac tumor found in children	Spider cell (vacuolated enlarged cardiac myocyte with clear cytoplasm due to abundant glycogen)	Bright echogenic ventricular mass, either protruding into the chamber or completely embedded in the wall	Isointense in T1, isointense to hyperintense in T2 No/minimal enhancement with contrast
	*Hemangioma*	2% of primary cardiac neoplasms	Variably sized blood vessels (capillary, cavernous or arteriovenous)	Hyperechoic lesion, in the 75% of case with an intramural growth and in the 25% of cases projecting in the cavity mimicking myxoma	Heterogeneous and hyperintense in T1 and T2 High and prolonged enhancement with contrast
The valves	*Fibroelastoma*	The most common valvular mass, particularly in aortic valve	Avascular fibroelastic fronds, endothelial lining, frequently entrapped thrombus	Usually pedunculated with a homogeneous speckled pattern and characteristic stippling along the edges	Very small and mobile
The conduction system	*Cystic tumor of the atrioventricular node*	Very rare	The cysts are filled by a mucoid substance and are lined by epithelium, cytokeratin and epithelial membrane antigen positive		
The pericardium	*Malignant mesothelioma*	2:1 male-female ratio	-Fibrous (spindle cell), and biphasic (mixed) -Negative adenocarcinoma markers, such as carcinoembryonic antigen (CEA) and positive mesothelial markers	Pericardial effusion	
Metastases		9.1% of all malignant tumors	Infiltrating malignant cells, necrosis	Multiple lesions. Pericardial effusion	Hypointense on T1 Hyperintense on T2 Heterogeneous enhancement with contrast

## Treatment and Prognosis

Surgical removal of benign cardiac tumors or masses, even if small and incidentally discovered, should always be considered in the setting of left-sided and endocavitary lesions due to the embolic risk. For right-sided and asymptomatic benign cardiac tumors, in the absence of a patent foramen ovale or septal defects, strict echocardiographic follow-up can be employed. All symptomatic benign tumors should be surgically resected (only exceptions are rhabdomyomas, as they often spontaneously regress or treated with mTOR complex 1 inhibitor [52]; intramural angiomas that can respond to corticosteroids; and fibromas, when the mass is unresectable and arrhythmias are under control by antiarrhythmic therapy) [53]. A surgical exposure is a “conditio sine qua non” for wide resection around the base of the tumor to prevent recurrence. In case of endocavitary ventricular neoplasms, surgical approach is through an ipsilateral atriotomy, if the tumor is located in the ventricular inflow, or through aortic or pulmonary arteriotomy, if it is located in the ventricular outflow [21]. When the neoplasm is intramural in the ventricles, ventriculotomy with mass enucleation is necessary. Most benign tumors can be resected *en bloc*, but in case of an unresectable tumor, a debulking is considered [21]. Orthotopic cardiac transplantation has been accomplished in the absence of metastasis [53].

The gold standard treatment for cardiac myxoma and lipoma is prompt surgical excision by experienced surgeons and complete removal of the entire base of the tumor. This approach should result in excellent early and long-term outcomes [5].

Potential of recurrence is typical for hemangiomas, and periodic echocardiography is recommended to examine for recurrence.

Cardiac myxomas seem to recur more often in young males, in patients with multifocal origins, in those who have a family history and in the setting of Carney complex [13••]. Patients should be followed up with TTE 1 year after surgery and every 5 years thereafter [15].

The standard treatment of fibroelastoma is surgical excision with either reconstitution or, less commonly, replacement of the valve. The root of the pedicle and the full thickness of endocardium involved is excised. Surgery is clearly indicated for patients who have had embolic events, complications that are directly related to tumor mobility (e.g., coronary ostial occlusion), and those with highly mobile or large (≥ 1 cm) tumors [13••].

Although complete resection is the treatment of choice for sarcomas, most patients develop recurrent disease and die even if their tumor can be completely resected. In patients with a limited tumor, complete resection with valve reconstruction or replacement when necessary can be undertaken and can improve long-term survival. The largest surgical cohort consists of 34 patients treated at the Mayo Clinic over a 32-year period. The median survival was significantly longer when a complete surgical resection was possible (17 vs 6 months when complete resection was not possible) [5]. As a general rule, resection should be attempted if feasible, provided the patient has acceptable performance status and no or limited metastatic disease. Patients who were able to undergo complete surgical resection had a median survival of 15 months, compared with only 5 months for those who were not eligible in a series reported by Simpson et al. [5].

The benefit of adjuvant chemotherapy and/or radiation is unknown but is offered to many patients, especially those with incomplete resections.

The overall survival of patients with malignant cardiac tumors remains poor [5]. The median survival as reported in prior studies ranged from 6 to 18 months.

Patients with cardiac angiosarcoma have the least favorable outcome. Angiosarcomas are associated with poor prognosis (median survival of 5 months versus 17 months for all other cardiac sarcomas), especially in case of metastatic disease at presentation when survival might be even lower (weeks). When the disease is isolated to the heart, surgical debulking might have some prognostic advantage [9]. Systemic therapy for cardiac angiosarcoma has been particularly disappointing, with cases of responses rarely reported [9].

## Conclusions

In the complex and heterogeneous field of cardiac masses, a proper differential diagnosis is extremely important in order to start the appropriate treatment. Emerging imaging modalities such as CMR and combined PET and CT may increase the diagnostic yield in terms of sensitivity and specificity for characterizing the lesions. Although an epidemiological and multimodality imaging approach is useful, the definite diagnosis requires histologic examination in challenging scenarios, and histopathological characterization remains the diagnostic gold standard allowing to establish the histological characteristics, treatment, and prognosis ^57^. Advances in understanding of molecular mechanisms have resulted in novel medical therapies that obviate the need for surgery (i.e., everolimus to treat rhabdomyomas). As we move into the era of next-generation sequencing and precision medicine, our understanding of these lesions will undoubtedly improve.

## References

[1] International Agency for Research on Cancer. *WHO Classification of Tumours of the Lung, Pleura, Thymus and Heart* 4th edn (World Health Organization, 2015).

[2] Basso C, Valente M, Poletti A, Casarotto D, Thiene G (1997). Surgical pathology of primary cardiac and pericardial tumors. Eur J Cardiothorac Surg.

[3] Ekmektzoglou KA, Samelis GF, Xanthos T (2008). Heart and tumors: location, metastasis, clinical manifestations, diagnostic approaches and therapeutic considerations. J Cardiovasc Med (Hagerstown).

[4] Oliveira GH, Al-Kindi SG, Hoimes C, Park SJ (2015). Characteristics and survival of malignant cardiac tumors: a 40-year analysis of > 500 patients. Circulation..

[5] Simpson L, Kumar SK, Okuno SH, Schaff HV, Porrata LF, Buckner JC, Moynihan TJ (2008). Malignant primary cardiac tumors: review of a single institution experience. Cancer..

[6] Bussani R, De-Giorgio F, Abbate A, Silvestri F (2007). Cardiac metastases. J Clin Pathol.

[7] Burke AP, Virmani R (1993). Cardiac myxoma. A clinicopathologic study. Am J Clin Pathol.

[8] Acebo E, Val-Bernal JF, Gomez-Roman JJ, Revuelta JM (2003). Clinicopathologic study and DNA analysis of 37 cardiac myxomas: a 28-year experience. Chest..

[9] Kupsky DF, Newman DB, Kumar G, Maleszewski JJ, Edwards WD, Klarich KW (2016). Echocardiographic features of cardiac angiosarcomas: the Mayo Clinic experience (1976–2013). Echocardiography.

[10] ElBardissi AW, Dearani JA, Daly RC (2008). Analysis of benign ventricular tumors: long-term outcome after resection. J Thorac Cardiovasc Surg.

[11] Garson A, Smith RT, Moak JP, Kearney DL, Hawkins EP, Titus JL, Cooley DA, Ott DA (1987). Incessant ventricular tachycardia in infants: myocardial hamartomas and surgical cure. J Am Coll Cardiol.

[12] Nakagawa Y, Ikeda U, Hirose M, Ubukata S, Katsuki TA, Kaminishi Y, Saito T, Hironaka M, Izumi T, Shimada K (2004). Successful treatment of primary cardiac lymphoma with monoclonal CD20 antibody (rituximab). Circ J.

[13] •• Castrichini, et al. Atrial thrombi or cardiac tumours? The image-challenge of intracardiac masses: a case report. Eur Heart J-Case Rep. 10.1093/ehjcr/ytaa026**Findings from this case report underline the role of multimodality imaging in the differential diagnosis.** 10.1093/ehjcr/ytaa026 PMC7180525 32352050

[14] Jain D, Maleszewski JJ, Halushka MK (2010). Benign cardiac tumors and tumorlike conditions. Ann Diagn Pathol.

[15] Jain S, Maleszewski JJ, Stephenson CR, Klarich KW (2015). Current diagnosis and management of cardiac myxomas. Expert Rev Cardiovasc Ther.

[16] Anavekar NS, Bonnichsen CR, Foley TA, Morris MF, Martinez MW, Williamson EE, Glockner JF, Miller DV, Breen JF, Araoz PA (2010). Computed tomography of cardiac pseudotumors and neoplasms. Radiol Clin N Am.

[17] Motwani M, Kidambi A, Herzog BA, Uddin A, Greenwood JP, Plein S (2013). MR imaging of cardiac tumors and masses: a review of methods and clinical applications. Radiology.

[18] Burke A, Tavora FR, Maleszewski J J & Frazier AA *Tumors of the Heart and Great Vessels* Vol. 22 (American Registry of Pathology, 2015).

[19] Maleszewski JJ, Larsen BT, Kip NS, Castonguay MC, Edwards WD, Carney JA, Kipp BR (2014). PRKAR1A in the development of cardiac myxoma: a study of 110 cases including isolated and syndromic tumors. Am J Surg Pathol.

[20] Neuville A, Collin F, Bruneval P, Parrens M, Thivolet F, Gomez-Brouchet A, Terrier P, de Montpreville VT, le Gall F, Hostein I, Lagarde P, Chibon F, Coindre JM (2014). Intimal sarcoma is the most frequent primary cardiac sarcoma: clinicopathologic and molecular retrospective analysis of 100 primary cardiac sarcomas. Am J Surg Pathol.

[21] Maleszewski JJ, Anavekar NS, Moynihan TJ, Klarich KW (2017). Pathology, imaging, and treatment of cardiac tumours. Nat Rev Cardiol.

[22] Araoz PA, Mulvagh SL, Tazelaar HD, Julsrud PR, Breen JF (2000). CT and MR imaging of benign primary cardiac neoplasms with echocardiographic correlation. Radiographics..

[23] Leduc C, Jenkins SM, Sukov WR, Rustin JG, Maleszewski JJ (2016). Cardiac angiosarcoma: histopathologic, immunohistochemical, and cytogenetic analysis of 10 cases. Hum Pathol.

[24] Bruna J, Lockwood M (1998). Primary heart angiosarcoma detected by computed tomography and magnetic resonance imaging. Eur Radiol.

[25] Burke AP, Rosado-de-Christenson M, Templeton PA, Virmani R (1994). Cardiac fibroma: clinicopathologic correlates and surgical treatment. J Thorac Cardiovasc Surg.

[26] Luna A, Ribes R, Caro P, Vida J, Erasmus JJ (2005). Evaluation of cardiac tumors with magnetic resonance imaging. Eur Radiol.

[27] Tworetzky W, McElhinney DB, Margossian R, Moon-Grady AJ, Sallee D, Goldmuntz E, van der Velde ME, Silverman NH, Allan LD (2003). Association between cardiac tumors and tuberous sclerosis in the fetus and neonate. Am J Cardiol.

[28] Lamba G, Frishman WH (2012). Cardiac and pericardial tumors. Cardiol Rev.

[29] Kocabas A (2013). Cardiac rhabdomyomas associated with tuberous sclerosis complex in 11 children: presentation to outcome. Pediatr Hematol Oncol.

[30] Beghetti M, Gow RM, Haney I, Mawson J, Williams WG, Freedom RM (1997). Pediatric primary benign cardiac tumors: a 15-year review. Am Heart J.

[31] Kiaffas MG, Powell AJ, Geva T (2002). Magnetic resonance imaging evaluation of cardiac tumor characteristics in infants and children. Am J Cardiol.

[32] Fieno DS (2006). Cardiovascular magnetic resonance of primary tumors of the heart: a review. J Cardiovasc Magn Reson.

[33] Basso C, Rizzo S, Valente M, Thiene G (2012). Prevalence and pathology of primary cardiac tumours. Cardiovasc Med.

[34] Tamin SS, Maleszewski JJ, Scott CG, Khan SK, Edwards WD, Bruce CJ, Oh JK, Pellikka PA, Klarich KW (2015). Prognostic and bioepidemiologic implications of papillary fibroelastomas. J Am Coll Cardiol.

[35] Klarich KW, Enriquez-Sarano M, Gura GM, Edwards WD, Tajik AJ, Seward JB (1997). Papillary fibroelastoma: echocardiographic characteristics for diagnosis and pathologic correlation. J Am Coll Cardiol.

[36] Cameselle-Teijeiro J, Abdulkader I, Soares P (2005). A lfonsín-Barreiro N, Moldes-Boullosa J, Sobrinho-Simões M. Cystic tumor of the atrioventricular node of the heart appears to be the heart equivalent of the solid cell nests (ultimobranchial rests) of the thyroid. Am J Clin Pathol.

[37] Patel J, Sheppard MN (2011). Cystic tumour of the atrioventricular node: three cases of sudden death. Int J Legal Med.

[38] Sharma GM, Linden D, Schultz DS, Inamdar KV (2010). Cystic tumor of the atrioventricular node: an unexpected finding in an explanted heart. Cardiovasc Pathol.

[39] Moorjani N, Kuo J, Wilkins D (2004). Left atrial phaeochromocytoma. Heart..

[40] Cohen PS, Israel MA, Roth JA, Ruckdeschel JC, Weisenburger TH (1989). Biology and treatment of thoracic tumors of neural crest origin. Thoracic oncology.

[41] Jeevanandam V, Oz MC, Shapiro B, Barr ML, Marboe C, Rose EA (1995). Surgical management of cardiac pheochromocytoma. Resection versus transplantation. Ann Surg.

[42] Vigneswaran WT, Stefanacci PR (2000). Pericardial mesothelioma. Curr Treat Options Oncol.

[43] Naramoto A, Itoh N, Nakano M, Shigematsu H (1989). An autopsy case of tuberous sclerosis associated with primary pericardial mesothelioma. Acta Pathol Jpn.

[44] Thomason R, Schlegel W, Lucca M, Cummings S, Lee S (1994). Primary malignant mesothelioma of the pericardium. Case report and literature review. Tex Heart Inst J.

[45] Suman S, Schofield P, Large S (2004). Primary pericardial mesothelioma presenting as pericardial constriction: a case report. Heart.

[46] Butz T, Faber L, Langer C (2009). Primary malignant pericardial mesothelioma—a rare cause of pericardial effusion and consecutive constrictive pericarditis: a case report. J Med Case Rep.

[47] Nilsson A, Rasmuson T (2009). Primary pericardial mesothelioma: report of a patient and literature review. Case Rep Oncol.

[48] Wolver S, Franklin RW, Abbate A. ST-segment elevation and new right-bundle branch block: broadening the differential diagnosis. Int J Cardiol 2006. Published Online First. 10.1016/j.ijcard.2005.11.056 16413073

[49] Maleszewski JJ, Bois MC, Bois JP, Young PM, Stulak JM, Klarich KW (2018). Neoplasia and the heart. J Am Coll Cardiol.

[50] Bhattacharyya S, Khattar R, Senior R (2013). Characterisation of intra-cardiac masses by myocardial contrast echocardiography. Int J Cardiol.

[51] Hoshal SG, Samuel BP, Schneider JR, Mammen L, Vettukattil JJ (2016). Regression of massive cardiac rhabdomyoma on everolimus therapy. Pediatr Int.

[52] Coelho PN, Banazol NG, Soares RJ (2010). Long-term survival with heart transplantation for fibrosarcoma of the heart. Ann Thorac Surg.

[53] Garatti A, Nano G, Canziani A, Gagliardotto P, Mossuto E, Frigiola A, Menicanti L (2012). Surgical excision of cardiac myxomas: twenty years experience at a single institution. Ann Thorac Surg.

